# Super-resolution of nodal and paranodal disruption in anti-pan-neurofascin-associated autoimmune nodopathy

**DOI:** 10.3389/fimmu.2025.1540859

**Published:** 2025-02-20

**Authors:** Vinicius da Cruz Neris Geßner, Janis Theobald Linke, Thomas-Otavio Peulen, Luise Appeltshauser, Claudia Sommer, Dirk Brämer, Christian Geis, Katrin Gertrud Heinze, Kathrin Doppler

**Affiliations:** ^1^ Department of Neurology, University Hospital Würzburg (UKW), Würzburg, Germany; ^2^ Rudolf Virchow Center, Center for Integrative and Translational Bioimaging, Julius-Maximilians-Universität Würzburg (JMU), Würzburg, Germany; ^3^ Section Translational Neuroimmunology, Department of Neurology, Jena University Hospital, Jena, Germany

**Keywords:** autoimmune nodopathy, polyneuropathy, dSTORM, Caspr-1, pan-neurofascin, node of Ranvier

## Abstract

**Introduction:**

In autoimmune nodopathies, autoantibodies target the nodes of Ranvier, impairing saltatory nerve conduction. Understanding the impact of autoantibody binding on protein assembly is crucial for gaining insights into the pathogenicity of different autoantibodies. We investigated nodal, paranodal, and cytoskeletal axonal proteins in teased fibers from a sural nerve biopsy of a patient with anti-pan-neurofascin autoantibodies. Conventional diagnostic tools, including fluorescence microscopy, often miss subtle alterations at the ultrastructural level.

**Methods:**

We utilized direct stochastic optical reconstruction microscopy (dSTORM), a super-resolution fluorescence imaging technique, to assess the nanoscale architecture of nodal, paranodal, and cytoskeletal axonal proteins.

**Result:**

While conventional fluorescence microscopy revealed severe paranodal and nodal damage in 14% of the nodes, with 86% appearing normal at first glance, the super-resolved images revealed a decreased neurofascin-155 and Caspr-1 density, but preserved colocalization of these adhesion proteins in paranodes that initially seemed normal. At the nodes, sodium channel density and distribution remained intact, but neurofascin-186 density was reduced. Axonal beta-IV spectrin was altered only in severely damaged nodes. This indicates that axonal integrity is largely preserved, with a potentially reversible decrease in paranodal and nodal adhesion proteins in patients with nodopathy revealing subtle alterations in nodal integrity that are not apparent with conventional imaging.

**Discussion:**

These likely reversible changes may explain the rapid recovery seen in patients with anti-pan-neurofascin autoantibodies following autoantibody depletion. Conversely, the small percentage of severely and axonally damaged nodes may account for the residual symptoms experienced by most patients.

## 1 Introduction

Autoimmune nodopathies with anti-neurofascin-155, anti-caspr-1 and anti-contactin-1 autoantibodies are associated with distinct clinical features, including severe sensorimotor neuropathy, sensory ataxia, tremor, and a poor response to IVIg therapy (1). These antibodies target paranodal proteins essential for the maintenance of axo-glial junctions at the nodes of Ranvier, which are crucial for saltatory conduction in myelinated nerves (2). Paranodal and nodal proteins exhibit a characteristic periodic arrangement (3). Anti-pan-neurofascin (NF) autoantibodies that are targeted against the nodal and paranodal isoform of NF were reported to induce a more severe but monophasic course of disease (4–6). Histopathological studies, including electron microscopy, have demonstrated that patients with paranodal autoantibodies exhibit characteristic paranodal dissection, distinguishing them from macrophage-mediated segmental demyelination, as found in chronic inflammatory demyelinating polyradiculoneuropathy (CIDP) (7–10). This supports the notion of a distinct pathophysiology targeting the nodes of Ranvier and its adhesion molecules. Although evidence of axo-glial dysjunction at the paranodes exists, information on the underlying molecular changes is lacking, as electron microscopy studies cannot visualize adhesion proteins at a molecular level.

Direct Stochastic Optical Reconstruction Microscopy (*d*STORM) offers a resolution beyond the diffraction limit of conventional microscopy, enabling the detailed visualization of protein distributions and interactions at the nanoscale level. This allows for the analysis of nodal, paranodal and axonal architecture on a super-resolved level, as demonstrated recently by our group and others (11, 12).

By analyzing a sural nerve biopsy specimen of a patient with anti-pan-NF autoantibodies, who presented with the typical, very severe but monophasic disease course, we aimed to gain further insights into the pathophysiology of anti-pan-NF autoantibodies by visualizing paranodal and nodal abnormalities on a molecular level.

## 2 Materials and methods

### 2.1 Patient

Nerve biopsy material was obtained from a male patient who was diagnosed with anti-pan-NF-associated autoimmune nodopathy. The diagnosis was based on a typical clinical picture with tetraplegia, areflexia and cranial nerve involvement, requiring mechanical ventilation for several months, and detection of autoantibodies against NF155 and NF186 by enzyme-linked immunosorbent assay (ELISA) and cell-based assays with NF-transfected human embryonic kidney 293 cells and confirmation by nodal and paranodal binding to murine teased fibers as previously described (4). CSF protein was highly elelvated (>2000 mg/l), CSF cell count was normal. Nerve conduction studies of the tibial, peroneal, sural, ulnar and median nerve did not detect any recordable sensory or compound motor nerve action potentials. The patient exhibited a classic monophasic disease progression, with complete elimination of anti-pan-neurofascin autoantibodies following treatment with plasma exchange, rituximab, and bortezomib. This was accompanied by a gradual and steady improvement of symptoms. The nerve biopsy of the sural nerve was obtained for diagnostic work-up six months after the onset of symptoms according to standard surgical procedures. The study was approved by the Ethics Committee of the University of Würzburg (number 158/21).

### 2.2 Human and murine tissue samples and teased fiber preparation

Murine sciatic nerves were dissected from adult C57/Bl6 mice. Human biopsy samples and murine nerves were cut into 5-mm slices, fixed in 4% paraformaldehyde for 10 minutes, then washed three times for 5 minutes each in phosphate-buffered saline (PBS) (1 M, pH 7.4), and processed for further analysis as previously described (13):

The epineurium and perineurium were removed with fine forceps under a stereo microscope. The remaining nerve fascicles were teased into single fibers using fine forceps in phosphate-buffered saline (1 M, pH 7.4). The main purpose of this procedure was to expose single nerve fibers and/or fiber bundles for better staining and visualization.

The 24-mm glass coverslips (Carl Roth, Marienfeld - Germany) on which the fibers were teased for *d*STORM and fluorescence imaging received special treatment beforehand: they were washed in isopropanol for 15 minutes, plasma cleaned for 10 minutes, and coated with poly-D-lysin (1 mg/ml) for 1 hour at 37°C. After the procedure, the samples were stored at -20°C.

### 2.3 Immunofluorescence staining

The teased fibers were immunostained using primary antibodies against Caspr-1 (polyclonal rabbit IgG, 0.2µg/ml, AbCam, ab34151), PanNF (polyclonal chicken, IgY, 0.4µg/ml, R and D Systems, AF3235), beta-IV spectrin (polyclonal rabbit IgG, 5 µg/ml, Invitrogen, PA5-62972), beta-II spectrin (polyclonal rabbit IgG, 5 µg/ml, BD Biosciences, ab34151), and Pan-sodium (polyclonal mouse IgG, 0.2µg/ml, Sigma, S8809). Prior to staining, the teased fibers were pre-permeabilized and fixed with ice-cold methanol at -20°C for 10 minutes, then washed with PBS three times for 5 minutes each. Afterward, the samples were permeabilized with 0.3% Triton-X in PBS for 30 minutes. Subsequently, the samples were blocked in a solution of 1% BSA + 0.3% Triton-X in PBS for 1 hour. The samples were then incubated with primary antibodies overnight at 4°C.

Secondary antibodies, including anti-rabbit Alexa Fluor 647 (A647, polyclonal goat IgG, 10 µg/ml, Jackson, #711-605-152), anti-chicken Cy3 (polyclonal goat IgG, 5 µg/ml, Jackson, 15914), and anti-mouse Alexa Fluor 488 (A488, polyclonal goat IgG, 5 µg/ml, Invitrogen, #A-110019), were used. The incubation with secondary antibodies was performed overnight at 4°C. For conventional fluorescence imaging, the teased fibers were embedded in a Vectashield antifade mounting medium (Vector Laboratories, VEC-H-1000). For *d*STORM, the samples were immersed in PBS until imaging in the *d*STORM-specific imaging buffer, inducing fluorophore blinking.

### 2.4 Super-resolution microscopy (*d*STORM) & image analysis

Dual-color *d*STORM microscopy was performed using an inverted light microscope (Zeiss Observer Z.1, Carl Zeiss AG) customized for single-molecule localization microscopy (SMLM) (**Supplementary Table 1**). The *d*STORM imaging buffer contained 125 mM cysteamine hydrochloride (Sigma, M6500), 20 mM D-glucose (Sigma, G7528), 0.55 mg/ml glucose oxidase (Roth, 60,281), and 0.011 mg/ml catalase (Sigma, C1345) in PBS at pH 7.7. The desired pH (7.7) was achieved using a 5M KOH solution. The dual-color *d*STORM images were acquired sequentially as summarized in **Supplementary Table 2**. Reconstruction of *d*STORM images was performed by ImageJ plugin ThunderSTORM (14, 15) and resulted in super-resolved images with a pixel size of 102 nm. For more details about the reconstruction process see **Supplementary Table 2**.

Autocorrelation analysis was performed on selected regions of interest (ROIs) to determine periodic protein arrangements as described previously (11) using the respective MATLAB script. Several regions of interest (ROIs) within the paranodal and internodal regions were manually selected in the super-resolved reconstruction of the respective node. The lengths along the axon were determined by selecting a start and end point of the centerline. Super-resolved images of Caspr-1, PanNF, beta-II and -IV spectrin, and Voltage-Dependent Sodium Channels (PanNaV+) were analyzed. Due to severely damaged ultrastructures, the periodicities from PanNF and Caspr-1 could not be retrieved and therefore not be quantified. All images with clear periodic structures were included in the peak-to-peak autocorrelation analysis, while those with low resolution or undefined structures were excluded.

Colocalization analysis was performed on the super-resolved images stained for Caspr-1 (to define the paranodal region) and stained for PanNF to visualize two isoforms of NF (-155 and -186) as previously described (11). For featuring the colocalization, we assessed the Manders A and B correlation coefficients (16, 17).

### 2.5 Paranodal and nodal protein density analysis by protein localization events in *d*STORM

The *d*STORM images of structural proteins from the para- and nodal regions of the nodes of Ranvier (specifically Caspr-1 and PanNF), from the patient under study and the mouse control, were compared by the number of localization events acquired over 20,000 frames (20 ms per frame) within the selected ROI. From a single node, a total of 3 ROIs each for Caspr-1 and PanNF were analyzed for patient and mouse control. The average localization events/µm^2^ were calculated to provide a relative measure of protein density. It is important to note that all parameters for *d*STORM acquisition and image reconstruction, as well as the concentration of antibodies for IHC and the methodology, were kept constant to allow for quantification (18).

### 2.6 *d*STORM image entropy analysis

SMLM images of nodes of Ranvier were analyzed using Shannon entropy as a metric for the nodes by their structural order. Nodes were automatically extracted based on their typical size using Gaussian filtering and Otsu’s thresholding. Shannon entropy was calculated for each node to quantify its complexity and information content This automated analysis enabled a comparison of nodal patterns with those of mouse controls, offering valuable insights into structural differences and organization. It also provided the analytical foundation for identifying and explaining the complete loss of periodicity in some samples (**Supplementary Figure 1**)

### 2.7 Morphometry and categorization of nodal damage by conventional high-content fluorescence microscopy

**Figure 1:**
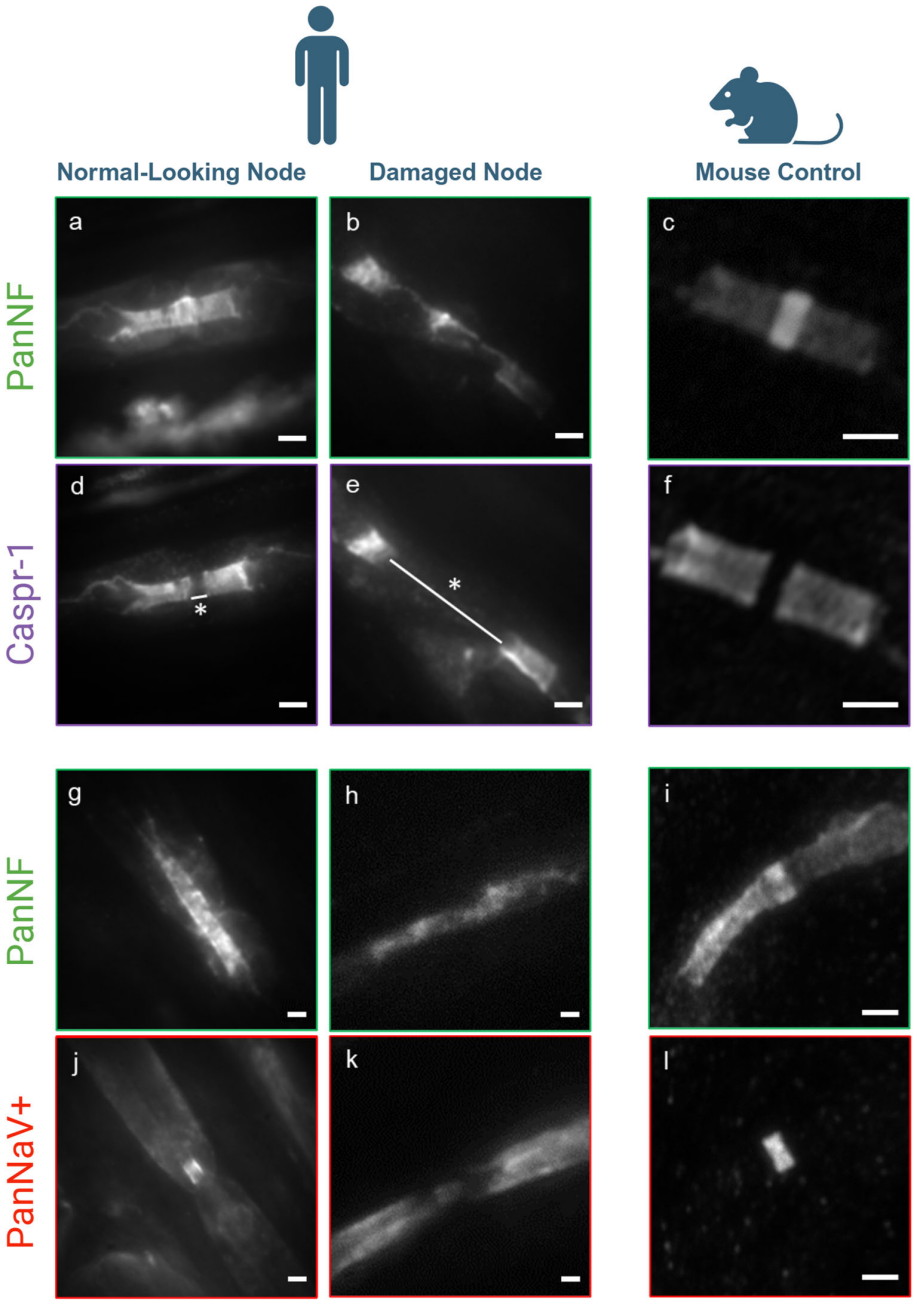
Conventional fluorescence microscopy image of nodal damage - Fluorescence images of PanNF, Caspr-1, and Pan sodium channels (NaV+) illustrate normal nodal architecture in the majority of nodes **(A, D, G, J)**, but severe destruction in several nodes **(B, E, H, L)** compared to a mouse control assessed by conventional fluorescence microscopy **(C, F, I, M)**. Patient (left): No damage to the structural proteins at the node of Ranvier is observed, with PanNF [green, (**A**, **G**)], Caspr-1 [purple, (**D**)], and Pan sodium channels (NaV+) [red, **J**)]. Normal nodal length (white line with star: 0.734 µm). Patient (middle): Severe damage to the structural proteins of PanNF [green, (**B**, **H**)], Caspr-1 [purple, (**E**)], and Pan sodium channels (NaV+) [red, (**L**)], with significant elongation of the nodes (white line with star: 4.04 µm). Mouse Control (right): **(C, F, I, M)** shows intact nerve structure for comparison. Pictograms created with BioRender.com (Scientific Image and Illustration Software). Scale bars: 1 µm.

High-content images (large field-of-view) (Leica DMi8 microscope, magnification of the objective lens: 20 x) were used to obtain a general overview of the nodes of Ranvier. (**Supplementary Figure 2**). Nodes were categorized into severely damaged nodes if the nodal architecture was apparently disrupted, with dispersion of Caspr-1 beyond the paranodes, severe elongation of the nodal gap and/or disruption of the staining of nodal/paranodal proteins, and normal-looking nodes if there was no obvious paranodal and nodal disruption and a clear block-like staining of anti-Caspr-1 and anti-pan-NF could be identified, together with a small anti-Nav-positive nodal gap (**Figure 1**). The percentage was calculated based on the number of normal-looking and severely damaged nodes over the total number of nodes in the image. A total of nine images were analyzed. For morphometric analysis, the length of the nodal gap, i.e. the distance between the paranodes as visualized by immunofluorescence staining with anti-Caspr-1, was measured. This was done manually using previously established methods (9).

### 2.8 Statistical analysis

Statistical analysis was performed using Origin Pro software (Origin2021b, OriginLab Corp.). Normality was tested with the Shapiro-Wilk test and the comparison of nodal length between the groups (murine nodes, normal-looking human nodes, severely damaged human nodes) was performed using Welch’s test. Results with *p*-values < 0.05 were considered significant.

## 3 Results

### 3.1 Only a small fraction of nodes of Ranvier looks abnormal by conventional fluorescence microscopy

**Figure 2:**
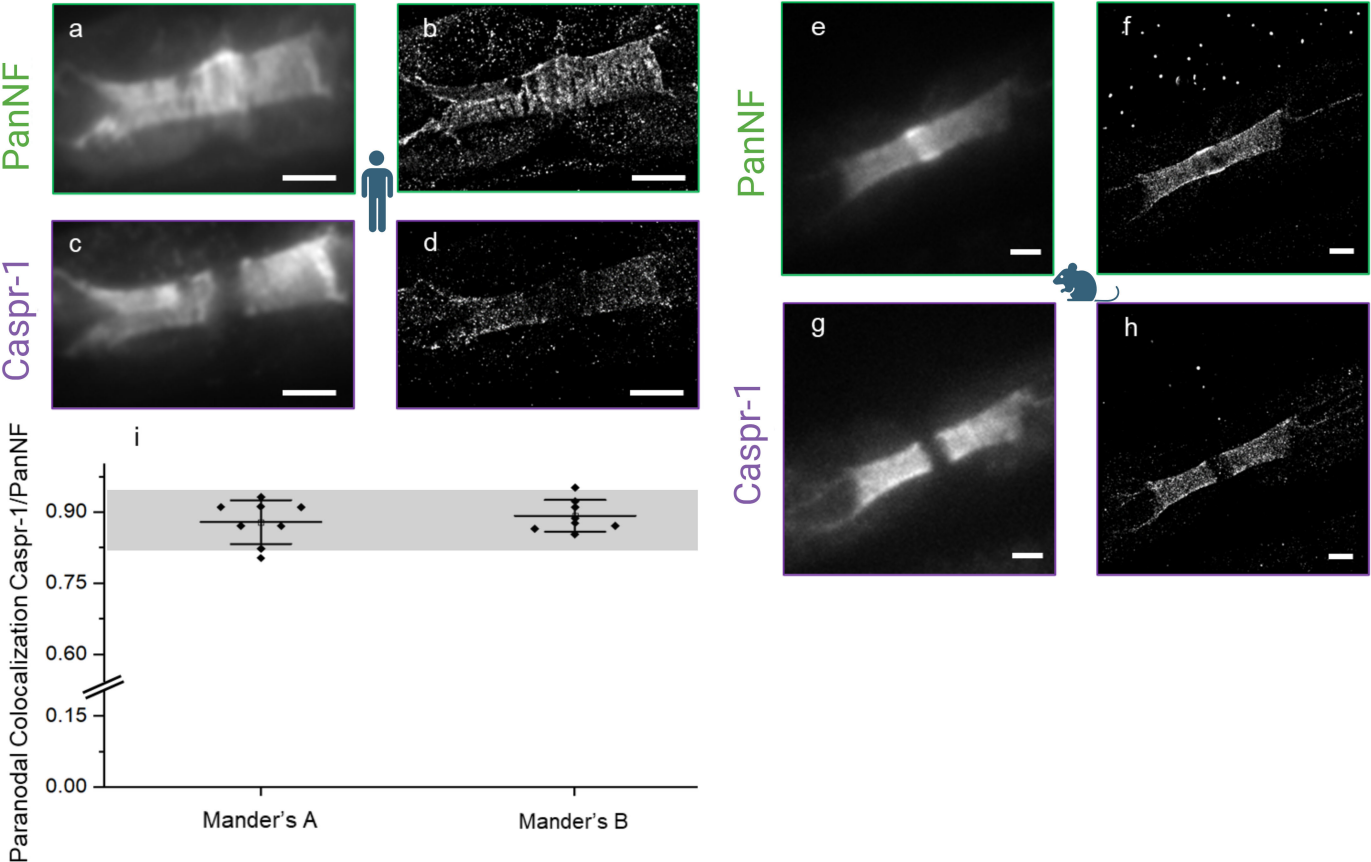
Damage at the node of Ranvier shown by conventional **(A, C, E, G)** versus super-resolved **(B, D, F, H)** fluorescence images (dSTORM) of PanNF and Caspr-1 at the node of Ranvier - Patient’s PanNF [green, (**A**)] and Caspr-1 [purple, (**C**)] and respective super-resolved images **(B, D)**, indicating damage of the node of Ranvier to the extent of the loss of their periodic characteristics. Mouse as reference [right, (**E–H**)] shows intact nerve structure for comparison. Colocalization analysis of Caspr-1 and NF-155 **(I)** indicates a synergistic damage effect in this autoimmune-mediated condition with Manders A (Colocalization Analysis of Caspr-1 over NF-155) and Manders B (Colocalization Analysis of NF-155 over Caspr-1). The grey bar represents the colocalization analysis coefficient range previously performed on mouse controls (11) and the black dots represent each ROI measurement. Pictograms from BioRender.com (Scientific Image and Illustration Software). Scale: 1 µm.

To evaluate the extent of damage to the nodes of Ranvier, we conducted a comprehensive assessment of a substantial number of nodes (n=100) using high-content imaging with anti-pan-NF and anti-Caspr-1 staining, following previously established protocols (11). Our analysis revealed that 14% of the nodes exhibited a significantly increased nodal length (mean: 3.16 ± 0.67 µm) compared to the normal values reported in a prior study (mean: 0.9 ± 0.3 µm) (11), with this difference being statistically significant (*p* = 2.10 x 10^-10^) (**Table 1**). These elongated nodes also demonstrated considerable disruption of the nodal protein architecture (**Figures 1B, E**). In contrast, the majority of the nodes (86%) maintained a normal architecture and length (**Figures 1A, D**) (mean: 0.943 ± 0.14 µm). We further refer to ‘normal-looking’ nodes as those with preserved nodal architecture, characterized by block-like staining of the paranodes with anti-Caspr1, a clearly identifiable nodal gap positively stained for Nav. Conversely, ‘abnormal-looking’ nodes exhibit disrupted nodal architecture, with paranodal protein staining dispersed along the fiber and/or a nodal gap that is significantly enlarged or absent (see **Figure 2** for an example).These two populations were compared qualitatively as well to a mouse control as shown in **Figures 1C**, **F**. Normal- and abnormal-looking nodes occurred within the same nerve fiber.

**Table 1:** Length of the nodal gaps in µm.

Mouse Control		Normal-looking Node		Damaged Node	
Mean	Standard Deviation	Mean	Standard Deviation	Mean	Standard Deviation
0.901	± 0.33	0.934	± 0.14	3.135**	± 0.60

Welch`s test was performed for all the 3 groups. No significant difference was found between wildtype mouse control (1) and samples with normal-looking nodes. The nodal length of damaged nodes was significantly increased compared to nodes of murine teased fibers.

**p<0.001.

Furthermore, sodium channels (NaV+) were assessed in both populations: normal-looking and severely damaged nodes. Qualitatively, the normal-looking nodes exhibited intact sodium channel integrity (**Figure 1J**) and normal architecture of PanNF proteins (**Figure 1G**). However, in the severely damaged nodes, this integrity was lost for both the sodium channels and the PanNF proteins (**Figures 1L**, **H**, respectively). These qualitative observations were compared with the mouse control, stained for sodium channels and PanNF protein, as shown in **Figures 1M**, **I**.

### 3.2 Loss of density and periodic arrangement of paranodal and nodal proteins is revealed by *d*STORM imaging

By *d*STORM we investigated subtle changes in the normal-looking nodes and evaluated nodal architecture at the nanoscale. We assessed 20 nodes using our custom *d*STORM setup and compared the results with data from a reference mouse control cohort (11). Our analysis revealed a qualitative decrease in the density of the paranodal proteins Caspr-1 and NF (NF-155) and nodal protein (NF-186) (**Figures 2A–D**) in the normal-looking nodes. This decrease was quantified by analyzing the density of localization events over the *d*STORM image acquisition from the respective super-resolved images of the normal-looking nodes and the *d*STORM images of the same protein structures in a mouse control (**Figures 2E–H**). This decrease ranged from 0.106 (mouse control) to 0.052 (patient) for Caspr-1 and 0.32 (mouse control) to 0.024 (patient), for PanNF, with both units expressed as average localization events per frame/µm^2^ (**Table 2**).

**Table 2:** Protein density quantification from the paranodal and nodal protein through localization event measurements from *d*STORM frame acquisition.

Mouse Control		Patient		Mouse Control		Patient	
Caspr-1		Caspr-1		PanNF		PanNF	
Mean	Standard Deviation	Mean	Standard Deviation	Mean	Standard Deviation	Mean	Standard Deviation
0.106	± 0.032	0.052	± 0.037	0.32	± 0.12	0.024	± 0.006

Unit – Average Number of Localization Events per frame/µm^2^

Despite the decrease, the colocalization of these proteins was preserved, indicating no axoglial disjunction (**Figure 2I**).

The periodicity of anti-Caspr-1 and anti-NF staining, however, was severely impaired and could not be quantified (**Figures 2B, D**). Further entropy analysis (Shannon entropy analysis) was done to confirm the total loss of the periodicity compared to a mouse control (**Supplementary Figure 1**).

**Figure 3:**
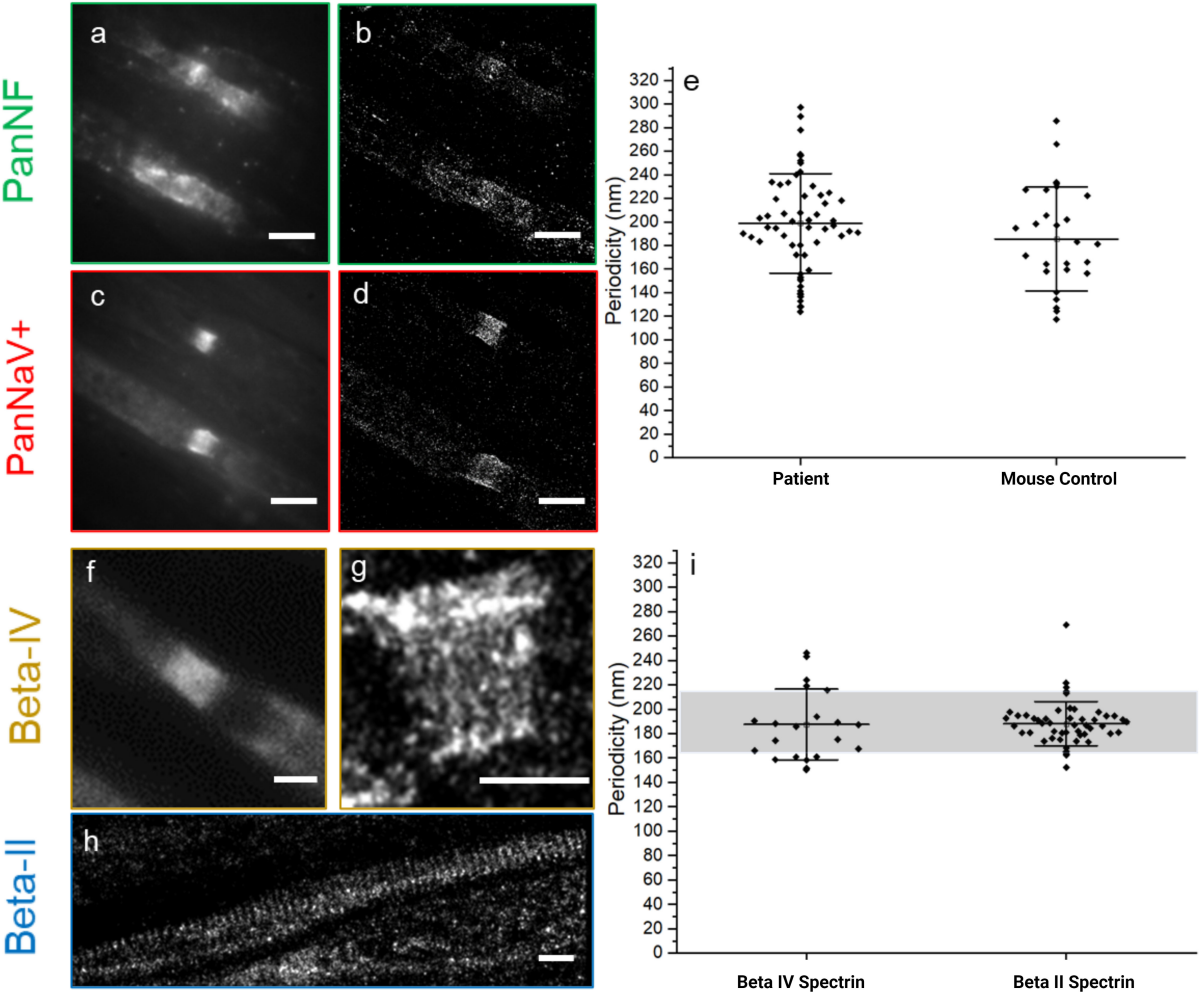
Integrity of the nodal/axonal cytoskeleton and sodium channels shown by conventional **(A, C, F)** versus super-resolved **(B, D, G, H)** fluorescence images (dSTORM) of PanNF, Pan sodium channels (PanNaV+), and Beta-IV and -II Spectrin - The auto-immune mediated damage, caused by autoantibodies against NF isoforms, is shown in human teased nerve fibers of a diagnosed patient **(A–D, F–H)**. PanNF [green, (**A, B**)] and Pan sodium channels (NaV+) [red, (**C, D**)] show damage to PanNF but minimal impact on voltage-dependent sodium channels. Quantification of the periodic arrangement of the Pan sodium channels (NaV+) **(E)** derived from the super-resolved images **(D)** and the mouse as reference indicate non-significant difference of the periodicity of the structures. Patient Nodal/axonal cytoskeleton was accessed, with beta-IV spectrin **(F, G)** and beta-II spectrin **(H)**, show that these structures remain intact, as confirmed by quantification of periodic characteristics **(I)**. The grey bar indicates the value of the mouse reference for beta-IV and beta-II spectrin based on measurement done for this work and previous publication from our group (11). The black dots represent each ROI measurement for periodicity. All the displayed periodic quantification is from the Patient under the study, except when it is written mouse control at x-axis **(E)**. Scale bars: 1 µm.

In contrast, the nodal sodium channels exhibited their characteristic periodicity (mean value: 198 ± 42 nm) (**Figures 3C, D, E**), despite the complete loss of periodicity in nodal (NF-186) and paranodal (NF-155) Neurofascin (**Figures 3A, B**).

### 3.3 Preserved beta-IV and beta-II spectrin indicates intact cytoskeleton

To evaluate the potential destruction of cytoskeletal architecture at the nodes of Ranvier and internodes as a marker for axonal damage, the ultrastructure of beta-IV and beta-II spectrin (nodal and internodal, respectively) was analyzed by *d*STORM imaging (**Figures 3F–H**). We did not detect any abnormalities of the periodicities of beta-IV and beta-II spectrin (187 ± 29 nm, beta-IV and 188 ± 18, beta-II) (graph 3i) indicating preservation of the nodal and axonal cytoskeleton. The periodicity measurement of beta-II spectrin was compared with the mouse control cohort from (9) and the beta-IV spectrin was compared with a mouse control cohort analyzed for this project (**Supplementary Figure 3**). We did not find any significant differences between the patient and the mouse control cohorts (**Table 3**). However, in severely damaged nodes, decreased or even absent beta-IV spectrin staining was observed. (**Supplementary Figure 4**).

**Table 3:** Measurement of nodal and axonal cytoskeleton protein periodicity in nm.

Mouse Control (10)		Patient		Mouse Control		Patient	
Beta-II		Beta-II		Beta-IV		Beta-IV	
Mean	Standard Deviation	Mean	Standard Deviation	Mean	Standard Deviation	Mean	Standard Deviation
184	± 20	188	± 18	186	± 24	187	± 29

A comparison between patient and mouse control cohorts from previous studies and measurements performed in this study. Welch`s test was performed for all the 3 groups. No significant difference was found between mouse control and the Patient measurement.

## 4 Discussion

By analyzing the nodal architecture in a sural nerve biopsy of a patient with pan-anti-NF-associated peripheral neuropathy, we demonstrate that *d*STORM imaging of paranodal and nodal proteins has the potential to reveal damaged nodal architecture even in nodes that appear normal by conventional fluorescence microscopy.

Thus, *d*STORM imaging of nodes of Ranvier is a potential tool to unravel nodal and paranodal damage on an ultrastructural level, even in only mildly affected nerves, and to detect early signs of damage. Nodal and paranodal disruption has been reported in several types of peripheral neuropathies in previous studies (19–22), but so far nodal/paranodal changes have barely been linked to the underlying pathogenicity and/or etiology. One reason may be that pathological changes are not specific to distinct pathogenic mechanisms. Another reason is that information on a molecular level has been lacking. In a previous study, we established a protocol for *d*STORM imaging of nodes of Ranvier in teased nerve fibers (11) that we here applied to the biopsy of a patient with anti-pan-NF autoantibodies.

Our findings of severe nodal destruction in only a small fraction of nodes but decreased density of Caspr-1 and NF accompanied by a loss of periodicity but with preserved axonal cytoskeletal integrity give important insights into the pathogenic effects of anti-pan-NF autoantibodies.

In contrast to studies of nerve and skin biopsies of patients with anti-Caspr-1, anti-NF155, and anti-contactin autoantibodies, where the majority of nodes were reported to be destroyed (7, 13, 23, 24), only 14% of the nodes were severely affected in our patient. This may be due to differences between paranodal autoantibodies or may reflect the longer course of disease in patients with paranodal autoantibodies, who mostly show a more chronic course of disease, whereas anti-pan-NF autoantibodies are associated with a severe but monophasic course of disease (25). Previous electron microscopy studies of patients with anti-contactin-1 and anti-NF autoantibodies described axoglial disjunction as a prominent finding in more than half of the nodes, with impaired adhesion of anti-contactin-1 and anti-NF155 suspected to be the underlying cause of detachment of the myelin sheath from the axon (7, 8). Our *d*STORM data revealed a decreased density of NF and Caspr-1, indicating that axoglial disjunction may be due to a loss of adhesion molecules rather than just impaired adhesion of the paranodal complex due to autoantibody binding.

In line with this, recent passive transfer studies reported a loss of paranodal proteins in animals treated with anti-NF155 or contactin-1 (26–28) and raised the hypothesis that in patients with anti-NF155 associated neuropathy, a decrease of paranodal NF-155 may be an effect of decreased protein turn-over (26). Another explanation might be the internalization of surface proteins induced by cross-linking which was shown in other diseases where not only IgG4 autoantibodies but also additional IgG subclasses were present (29, 30). When incubating myelinated dorsal root ganglion neuron cultures with anti-NF155 and anti-pan-NF, damage to the paranodes was reported and more severe after incubation with anti-pan-NF compared to anti-NF155 (25). However, sodium channel density and periodicity were within the normal range in our patient. In a previous electron microscopy report of a patient with anti-pan-NF autoantibodies, loss of nodal microvilli but no paranodal abnormalities were described (31). However, the same author later described another case of anti-pan-NF autoantibodies where paranodal axoglial disjunction could be observed by electron microscopy, consistent with our *d*STORM data (32). The normal-looking paranodes in the first case may be explained by a relatively short course of disease and quick and complete recovery [in contrast to other known cases of anti-pan-NF-associated disease that were monophasic, but with a longer course of disease and slower recovery from residual symptoms (25)].

When studying sural nerve biopsies, as in our study, it needs to be taken into account, that only sensory nerve fibers are included. However, as neurofascin is equally found on motor nerve fibers, results can most probably be transferred to motor nerves.

**Figure 4:**
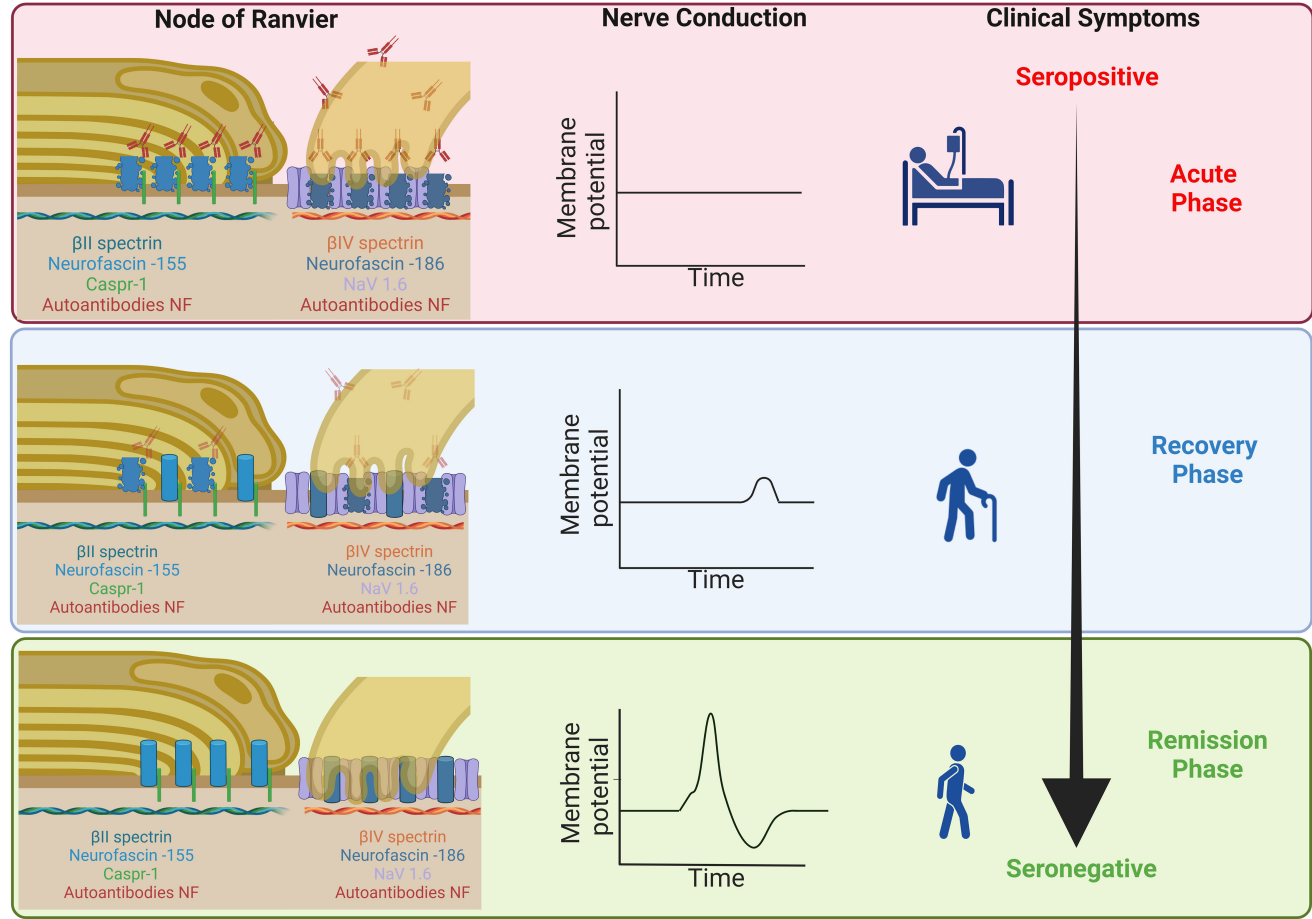
Schematic illustration of the hypothesized structural damage during the course of disease – During the acute phase of the disease, autoantibodies bind to nodal and paranodal NF, inducing a loss of the proteins of the paranodal complex and nodal NF and thus inducing axoglial dysjunction and conduction block but no axonal damage (upper image). During recovery, autoantibody titers decrease, the density of paranodal proteins normalizes (due to the natural protein turn-over) and patients recover (middle image). At remission, the density of the paranodal protein complex is normal and damage to the nodes completely reversed (lower image). Severe damage to a small percentage of nodes (including axonal damage) that may explain residual symptoms is not shown here. Made with BioRender.com (Scientific Image and Illustration Software).

Pathophysiologically, complete tetraplegia in patients with anti-pan-NF, followed by rapid recovery after a decrease of autoantibody titers as reported in the literature (5, 6, 33), can be explained by reversible conduction failure, possibly induced by dysfunction of the sodium channels at the nodes without any structural damage. In acute motor axonal neuropathy (AMAN), reversible conduction failure is explained by the temporary loss of nodal sodium channels (34). In animal models, anti-GM1 (anti-Monosialotetrahexosylganglioside 1) and anti-Gd1 (anti-Disialotetrahexosylganglioside 1) autoantibodies were shown to induce loss of nodal sodium channels (35, 36). In 20-40% of the nodes, disruption of beta-4 spectrin was observed after treatment with Gd1a (Disialotetrahexosylganglioside 1a), GT1a/2b (Trisialotetrahexosylganglioside 1a/2b) and Gd1b (Disialotetrahexosylganglioside 1b), thus also including the cytoskeleton (37). Complement deposition and cytotoxicity is supposed to play an important role in AMAN (38). In patients with anti-pan-NF autoantibodies, the clinical picture of patients with IgG1 and IgG3 autoantibodies (that both induce activation of complement) and IgG4 (that does not bind complement) is similar (25), arguing against a solely complement-mediated effect. Our data raise the hypothesis that tetraplegia in patients with anti-pan-NF-associated neuropathy is induced by autoantibody binding leading to temporary nodal dysfunction without any structural damage and immediate reversibility after a decrease of autoantibodies. Residual symptoms that improve within a delay of several months may be explained by paranodal damage that is reversible by the natural turnover of paranodal proteins (**Figure 4**). Long-term residual symptoms could be due to severe destruction of some nodes that was also demonstrated by our study indicated by decreased spectrin-IV staining (**Supplementary Figure 4**).

## 5 Conclusions

We demonstrated that *d*STORM imaging of nodes of Ranvier is a valuable tool for investigating nodal architecture on a molecular level, providing further evidence of paranodal disruption even in nodes that appear normal by conventional immunofluorescence microscopy. The preserved axonal cytoskeleton supports the notion of reversible conduction failure as the correlate of severe motor dysfunction and paranodal abnormalities as a potential cause of milder but longer-lasting symptoms. These findings need to be confirmed and further validated in larger patient cohorts.

## Data Availability

The original contributions presented in the study are included in the article/**Supplementary Material**. Further inquiries can be directed to the corresponding authors.

## Glossary

IVIg: Intravenous Immunoglobulin
dSTORM: Direct Stochastic Optical Reconstruction Microscopy
NF: Neurofascin
CIDP: Chronic Inflammatory Demyelinating Polyradiculoneuropathy
PBS: Phosphate-Buffered Saline
ROIs: Regions of Interest
NaV+: Voltage-dependent Sodium Channels
ELISA: Enzyme-Linked Immunosorbent Assay
JMU: Julius-Maximilians-Universität Würzburg
IZKF: Interdisciplinary Center of Clinical Research
SMLM: Single-Molecule Localization Microscopy
GSLS: Graduate School of Life Sciences
BSA: Bovine Serum Albumin
IgG: Immunoglobulin G
Caspr-1: Contactin-associated Protein-1
AMAN: Acute Motor Axonal Neuropathy
GM1: Monosialotetrahexosylganglioside 1
Gd1: Disialotetrahexosylganglioside 1
GD1a: Disialotetrahexosylganglioside 1a
GT1a/2b: Trisialotetrahexosylganglioside 1a/2b
Gd1b: Disialotetrahexosylganglioside 1b
