# Supplementary material for: Super-resolution of nodal and paranodal disruption in anti-pan-neurofascin-associated autoimmune nodopathy
Source: Front Immunol. 2025 Feb 20;16:1540859. doi: 10.3389/fimmu.2025.1540859 (PMC11882429; doi:10.3389/fimmu.2025.1540859)
Supplement: Supplementary file 1 [file DataSheet1.docx]

Supplement Table 1 - Home-made setup for dStorm and Fluorescence Images

Supplement Table 2 – Image process details and resolution from epifluorescence and SMLM images

**
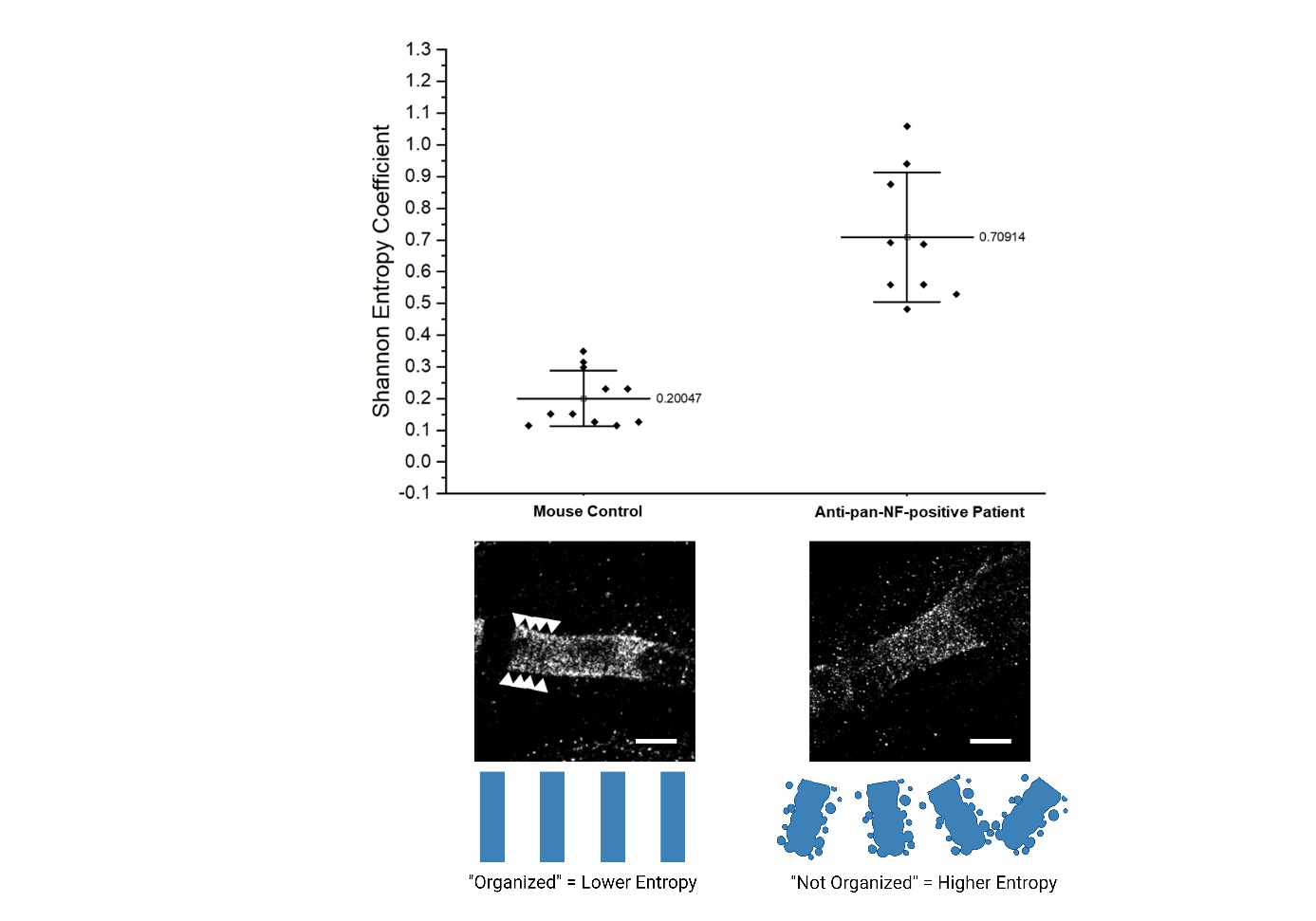
**

**Supplement Fig 1 - Shannon entropy coefficient calculation to determine** **the entropy level and thus the organization level of the super-resolved images of the nodal structural proteins**. In the case of Caspr-1 and PanNF the ultrastructural damage level was so prominent when observed by super resolution that the periodic characteristic was completely lost and could not be quantified. This is demonstrated at the organization level of Caspr-1 protein of mouse control (low entropy) and the anti-panNF-positive patient (high entropy).

Pictograms from BioRender (Scientific Image and Illustration Software).

Scale bar: 1µm


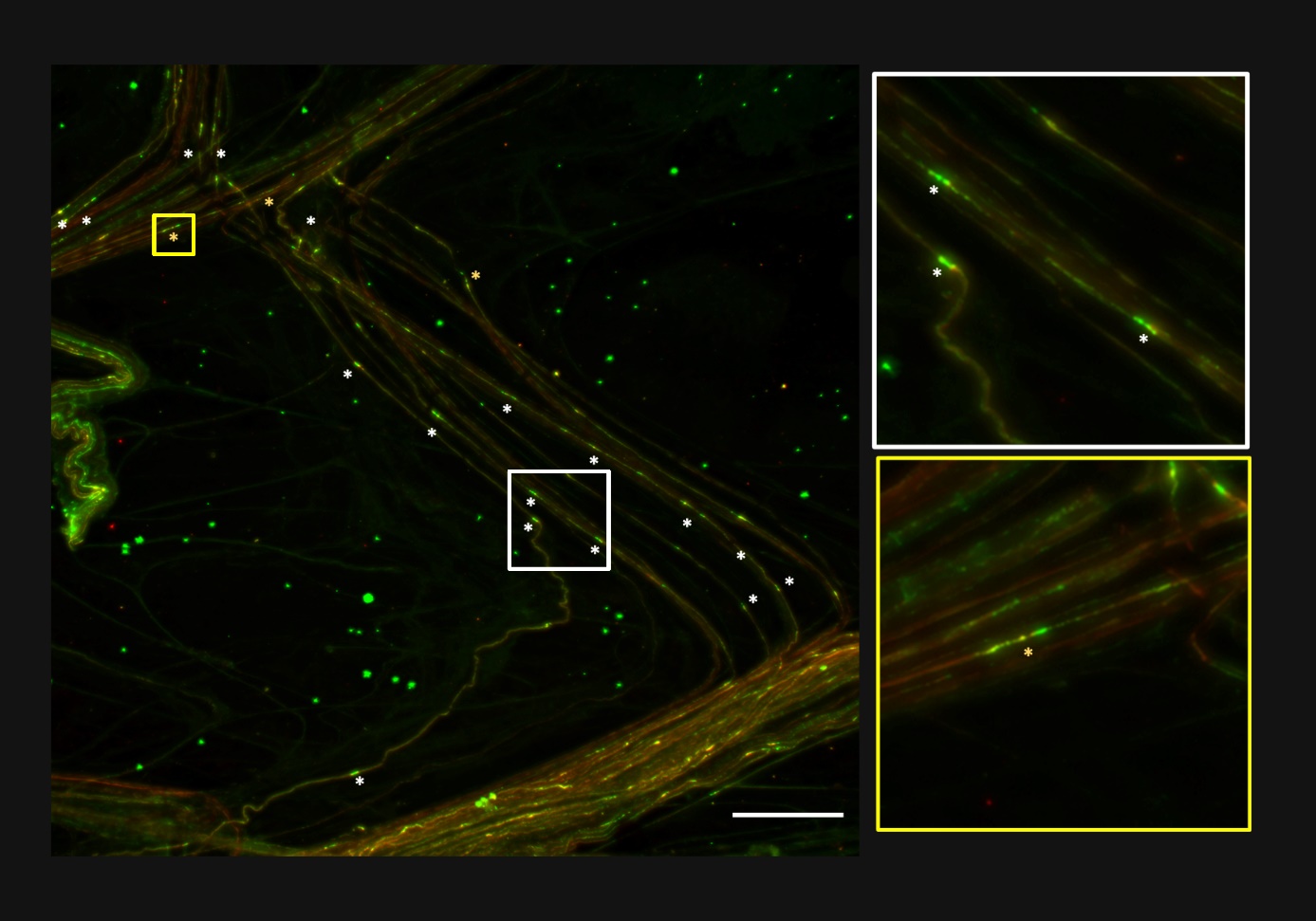


**Supplement Fig 2 – Epifluorescence overview image of teased fibers of the sural nerve biopsy of the anti-panNF-positive patient shows two populations of normal-looking or severely damaged nodes. The samples were stained for Pan NF (green) and Caspr-1 (red). White asterisks represent the normal nodes (not elongated). Yellow asterisk represents the damage nodes (eloganted). Quantification was done using 9 images and counting proportional population of the two populations in respect of the relative area of the image.**

Scale bar: 25 µm

**
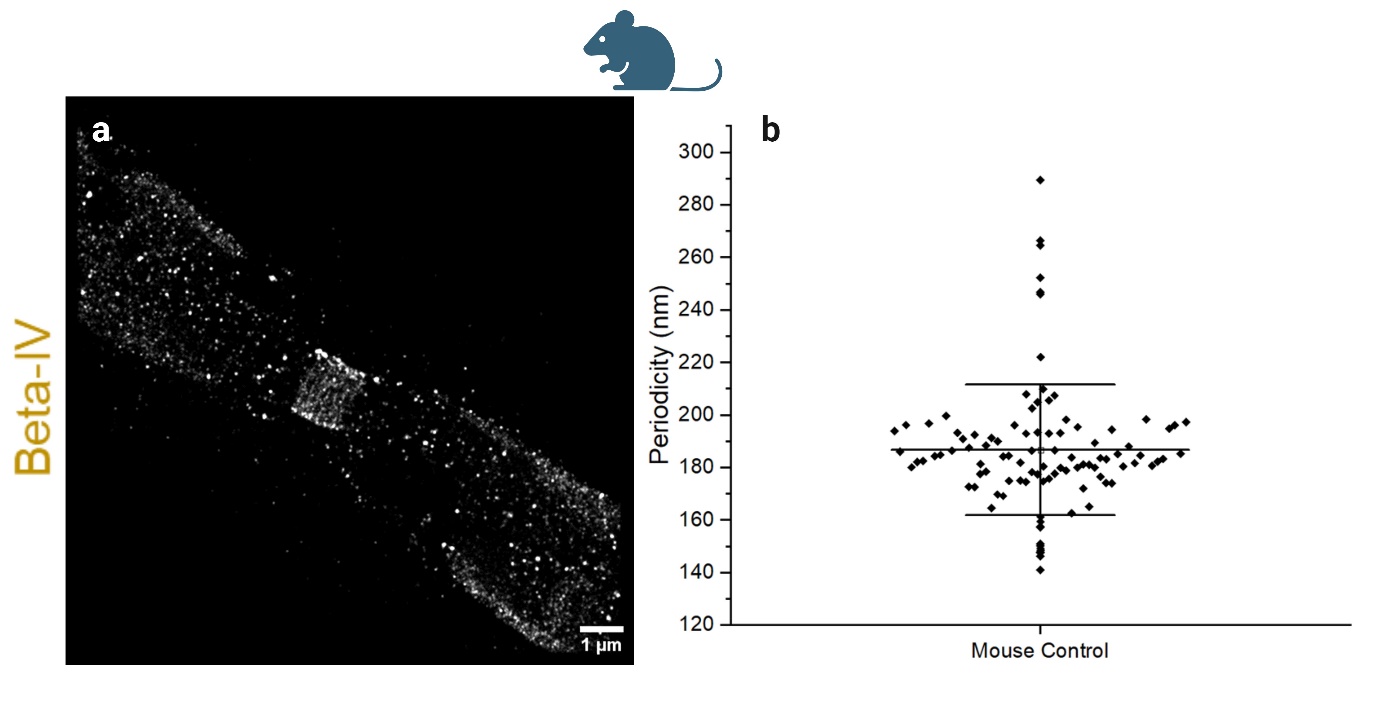
**

**Supplement Fig 3 - Quantification of the Periodic Structure of Beta-IV Spectrin in Super-Resolved Images of Sural Nerve Biopsies from Mouse Control.** This figure presents the super-resolution imaging of Beta-IV spectrin in sural nerve fibers from mouse control biopsies, obtained using dSTORM. Image of the periodic arrangement of Beta-IV spectrin along node of Ranvier is clearly visible, demonstrating its regular spacing (a). Quantitative analysis of the spatial distribution was performed, confirming the characteristic periodicity of approximately ~190 nm (186.72 ± 24.91), (n=5) (b). These findings provide a baseline for comparison with the patient.

**
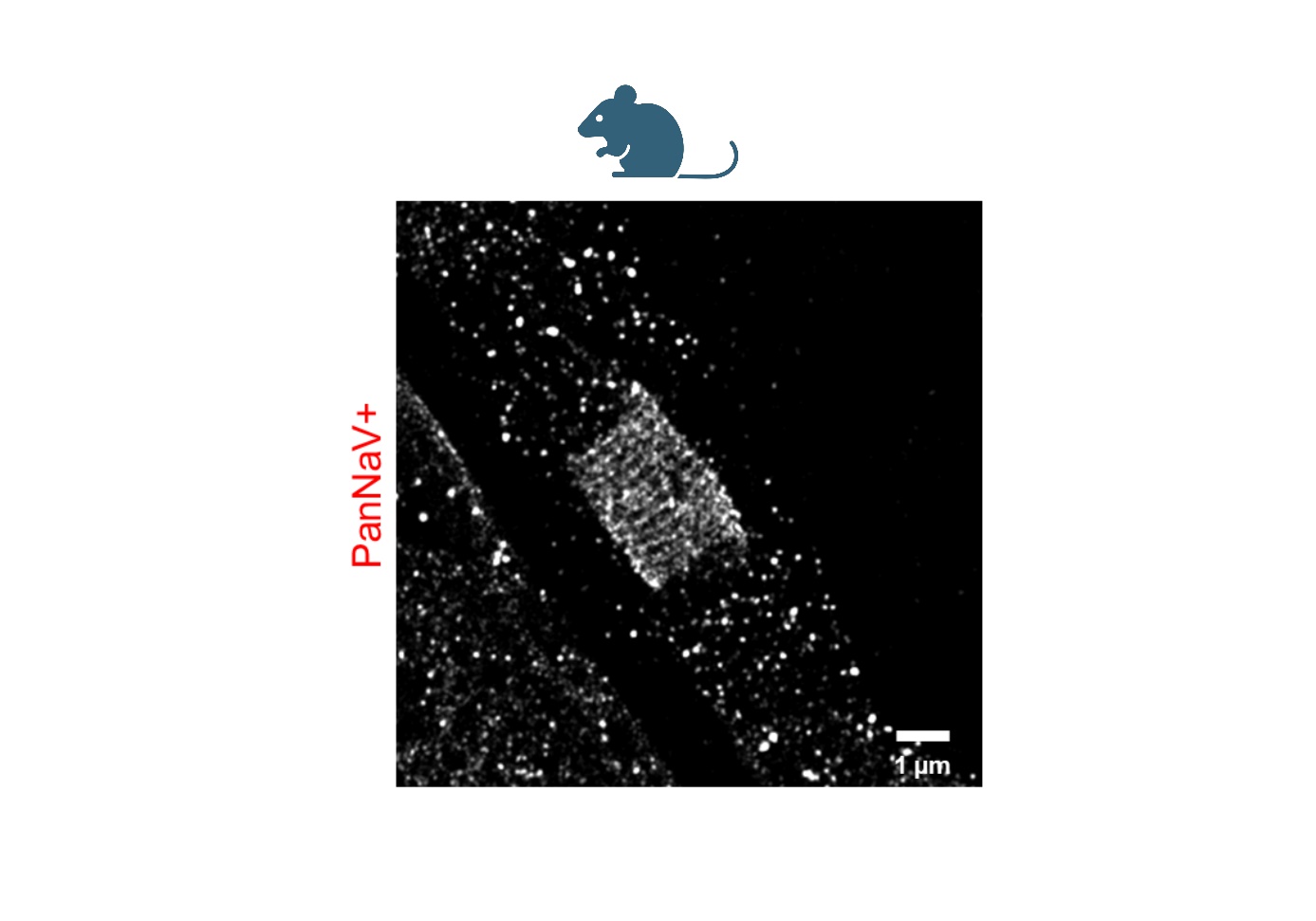
**

**Supplement Fig. 4 – Super-resolved image of PanNaV+ in sural nerve biopsies from mouse control used for periodic structure quantification.** This figure presents super-resolution imaging of PanNaV+ in sural nerve fibers from mouse control biopsies, obtained using dSTORM. The periodic arrangement of PanNaV+ along the node of Ranvier is clearly visible, demonstrating its regular spacing. These findings provide a baseline for comparison with the patient under study.

**
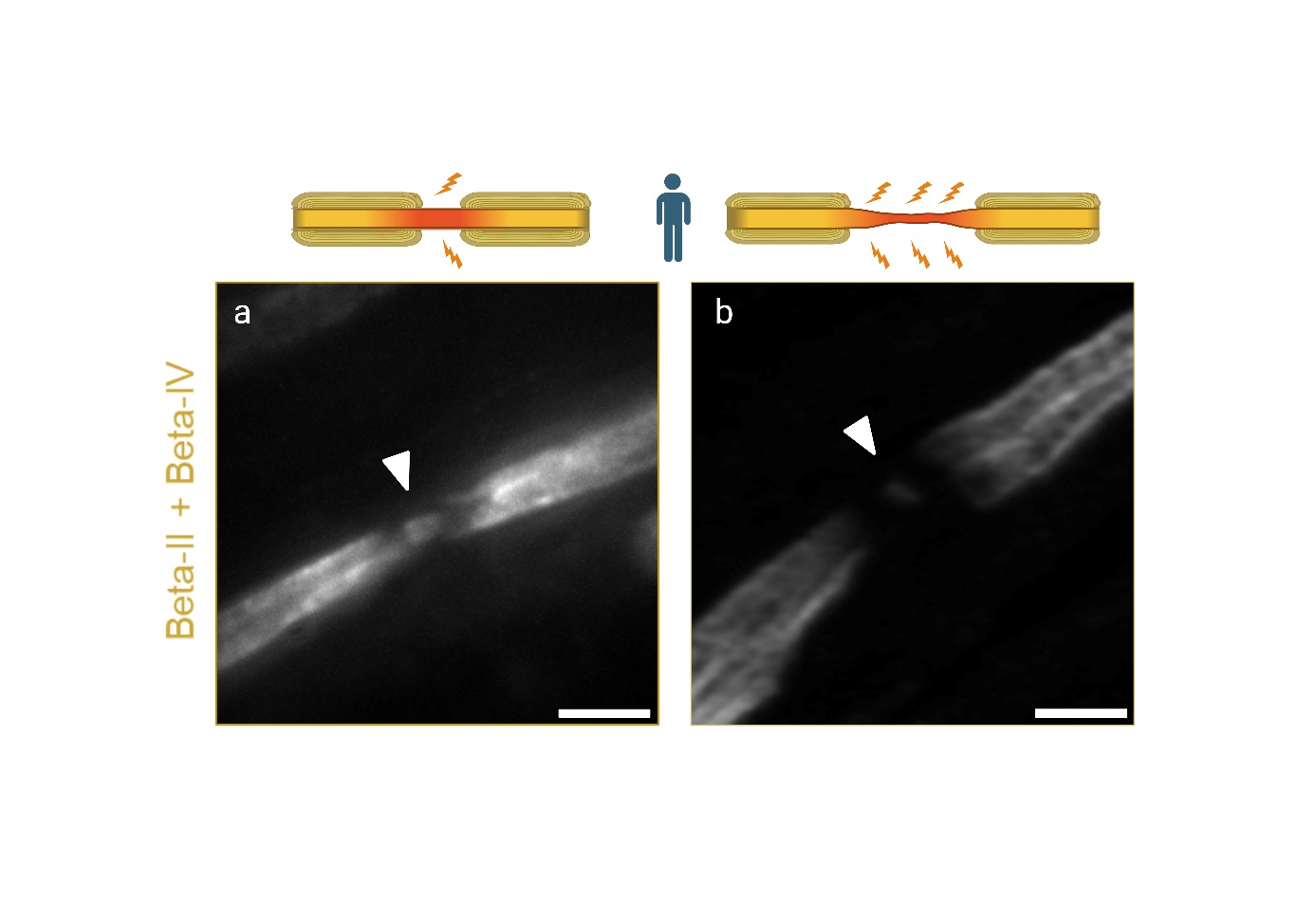
**

**Supplement Fig 5 - Nodal Length and the ultrastructural damage of the axonal cytoskeleton and structural proteins of the para- and nodal regions in “normal-looking” node (a) and severely damaged (b) nodes of a patient with pan-NF autoantibodies -** White arrowhead in (a) indicates that Beta IV spectrin is still preserved (nodal cytoskeleton preserved), and less present in (b) with severe nerve damage and diminished or no staining for Beta IV spectrin, suggesting damage at the nodal cytoskeleton. Pictogreams from BioRender (Scientific Image and Illustration Software).Scale bars: 5 µm.
